# Progenitor Cell Therapy to Treat Acute Myocardial Infarction: The Promise of High-Dose Autologous CD34^+^ Bone Marrow Mononuclear Cells

**DOI:** 10.1155/2013/658480

**Published:** 2013-04-28

**Authors:** Joseph C. Poole, Arshed A. Quyyumi

**Affiliations:** ^1^Emory University School of Medicine, Division of Cardiology, 319 Woodruff Memorial Research Building, 1639 Pierce Drive, Suite 319, Atlanta, GA 30322, USA; ^2^Emory Clinical Cardiovascular Research Institute, 1462 Clifton Road NE, Suite 506, Atlanta, GA 30322, USA

## Abstract

ST elevation myocardial infarction (STEMI) is associated with an increased risk for congestive heart failure and long-term mortality despite the widespread use of thrombolysis and catheter-based revascularization. The need for improved post-STEMI therapies has led to a surge of novel therapeutics, especially regenerative approaches using autologous mononuclear cells. Indeed, the past decade has been marked by a number of human trials studying the safety and efficacy of progenitor cell delivery in the post-STEMI setting. While a variety of cell types and delivery techniques have been utilized, directed therapy to the infarct-related artery has been the most widely used approach. From over 1300 subjects randomized in these studies, there is sufficient evidence to conclude that cell therapy after STEMI is uniformly safe, while the efficacy of this intervention for improving outcomes is less clear. Recent meta-analyses have highlighted the importance of both timing of cell delivery, as well as the type, quantity, and mobility of delivered cells as determinants of response. Here, we show the case in which higher doses of CD34^+^ cells, which are more potent in terms of their migratory capacity, offer the best hope for preserving cardiac function following STEMI.

## 1. Introduction

Despite early thrombolysis and revascularization, ST elevation myocardial infarction (STEMI) carries significant morbidity and mortality [1, 2]. Following acute STEMI, failure of prompt revascularization leads to myocardial necrosis that can cause ventricular chamber dilation through adverse remodeling, often leaving patients with permanent left ventricular (LV) systolic dysfunction and progressive congestive heart failure [3, 4]. Optimal medical therapy and cardiac rehabilitation in the postinfarct period helps minimize adverse remodeling; however, 12-month mortality for patients with STEMI and LV dysfunction still exceeds 10% [5]. 

In a landmark preclinical study, Orlic et al. demonstrated that direct injection into the infarcted myocardium of a highly defined bone marrow derived-cell (Lin^−^ c-kit^pos^) population with hematopoietic and endothelial progenitor potential improved morbidity and mortality in a murine MI model. Within 3–5 hours of an induced anterior MI, mice received either Lin^−^ c-kit^pos^ cells, Lin^+^ cells, or no injection. In animals receiving Lin^−^ c-kit^pos^ cells, more than two-thirds of the infarcted myocardium was repopulated with regenerated myocytes; there was clear neovascularization, and cardiac function improved.

The need for improved postinfarct therapies, together with the promise of regenerative medicine, has spawned a surge in human trials studying the safety and efficacy of progenitor cell delivery in the post-STEMI setting. While a variety of cell types and delivery techniques (intravenous, direct myocardial injection, nonspecific bone marrow stimulation and intra-arterial) have been utilized in the postacute MI setting, the majority of studies have used a percutaneous catheter-based approach to direct therapy to the coronary artery. To date, at least 17 randomized controlled trials (RCTs) have investigated infarct-related artery (IRA) infusion of bone marrow-derived mononuclear cells (BMMNCs) using the “stop-flow technique” [6] following acute STEMI (Table 1). From more than 1300 subjects randomized in these studies, there is sufficient evidence to conclude that cell therapy after STEMI is uniformly safe, while the efficacy of this intervention in improving left ventricular ejection fraction (LVEF) and major adverse cardiovascular events (MACEs) has been less clear. Subgroup analyses in recent meta-analyses have highlighted the importance of both timing of cell delivery, and the type, quantity, and mobility of delivered cells as determinants of response and specifically suggest that higher doses of CD34^+^ cells that are potent in terms of their migratory capacity offer the best hope for preserving cardiac function following STEMI [7–9]. 

## 2. Autologous Progenitor Cell Therapy after STEMI

The transplantation of progenitor cells and regeneration enhancement in acute myocardial infarction (TOPCARE-AMI) trial was the first randomized controlled trial (RCT) to demonstrate functional improvement by BMMNCs following STEMI [10]. In total, 59 subjects were enrolled in TOPCARE-AMI, 29 receiving BMMNCs, and 30 subjects receiving circulating mononuclear cells. Cells were delivered to the infarct-related artery at 4.9 ± 1.5 days after STEMI. At a mean followup of 4 months, LVEF improved in the BMMNC group from 49 ± 10% to 57 ± 10% (*P* < 0.001) and from 51 ± 10% to 59 ± 10% (*P* < 0.001) in the group receiving circulating progenitor cells. Subsequent followup indicated long-term safety of the therapy at 1 [11] and 5 years [12]. In a combined analysis of 55 subjects available for 5-year followup, LVEF by cardiac magnetic resonance imaging improved from 46 ± 10% to 57 ± 10% (*P* < 0.001) and functional infarct size similarly improved as measured by late enhancement volume normalized to LV mass (*P* < 0.001). 

In the Bone marrow transfer to enhance ST-elevation infarct regeneration (BOOST) trial, the efficacy of autologous BMMNCs delivered to infarct-related artery was evaluated in 60 patients 4.8 ± 1.3 days after STEMI [13]. In distinction to TOPCARE-AMI, the control group in the BOOST trial received optimum postinfarction medical therapy, but no cells. At 6-month followup, mean LVEF improved by 6.7% in the cell therapy group (from 46.3 ± 10.6% to 53 ± 15.5%) and by 1.1% in the control group (from 47.8 ± 9.7% to 48.9 ± 15.2%) (*P* value for between-group comparison = 0.04). Long-term followup of surviving subjects at 5 years (*N* = 56; mean followup 61 ± 11 months) revealed no difference in cardiac function or MACE between groups. LVEF assessed by magnetic resonance imaging decreased by 3.3 ± 9.5% in the control group and by 2.5 ± 11.9% in the group that received BMMNCs (*P* = 0.30) [14]. 

To date, three other studies comparing autologous BMMNCs to a placebo control have reported positive results [15–17]. The Reinfusion of Enriched Progenitor Cells and Infarct Remodeling in Acute Myocardial Infarction (REPAIR-AMI) trial is the largest trial to date to evaluate the efficacy of BMMNCs in the post-STEMI setting [17]. This multicenter trial randomized 204 subjects to receive either BMMNCs or placebo medium, from 3 to 7 days after STEMI. At 4-month followup, LVEF improved by 5.5 ± 7.3% in the treatment group and by 3.0 ± 6.5% in the placebo group (*P* = 0.01 between groups). At 2 years, the occurrence of MACE was significantly lower in the cell therapy group (28%) compared to placebo group (43%) (*P* = 0.025) [18]. The Finish stem cell study (FINCELL) randomized 80 subjects to receive intracoronary autologous BMCs or placebo media at 2–6 days after STEMI [16]. At six months of followup, the BMC treatment group had significant improvement in LVEF by transthoracic echocardiogram compared to the placebo group (4.0 ± 11.2 versus −1.4 ± 10.2%; *P* = 0.03). No differences in MACE at 6 months between groups were reported. Ge et al. reported similar findings in a smaller study (*N* = 20) in which the treatment group received autologous BMMNCs 12 hours after diagnosis of STEMI [15]. In the 10 subjects who received cells, LVEF improved at 6-month followup from 53.8 ± 9.2 to 58.6 ± 9.9; *P* < 0.05, but remained unchanged in the placebo group. The potential impacts of myocardial stunning in the immediate post-STEMI period (when cells were delivered), the small number of subjects randomized, and the relatively preserved LVEF immediately after STEMI make this study more difficult to interpret compared to REPAIR-AMI and FINCELL. 

Positive trials comparing autologous BMMNCs to placebo have been encouraging, but they must be interpreted with caution given that many trials have reported neutral findings [19–23]. Among these negative trials is the recently concluded LateTIME study that evaluated the efficacy of delaying delivery of autologous BMMNCs 2-3 weeks following STEMI [22]. The LateTIME trial was a multicenter US trial performed by the National Heart, Lung, and Blood Institute-sponsored Cardiovascular Cell therapy Research Network. Rationale for testing the benefit of later cell delivery was both practical and evidence based. Most patients with STEMI present to centers lacking expertise in cell therapy, and several trials have demonstrated that cell therapy preferentially benefits patients with lower LVEF [17, 24, 25] who may be too sick or unstable to tolerate bone marrow aspiration and cell delivery in the first few days following STEMI. A total of 87 patients were randomized to receive either autologous BMCs or placebo. At 6-month followup, there was no significant difference between treatment and placebo arms for either primary (changes in LVEF and regional wall motion in the infarct and border zones) or secondary outcomes (changes in LV volumes and infarct size). 

## 3. Justification for Using Purified Cells with Increased Regenerative Potential

Given disparate findings from a decade of RCTs, the efficacy of intracoronary infusion of autologous BMMNCs has been evaluated by several meta-analyses [7–9]. These analyses demonstrate that BMMNC therapy is safe and associated with modest improvement in LVEF (3.66% absolute improvement), reduction in infarct scar size (−5.49%), and reduction in LVESV (−4.80 mL). Subjects receiving BMMNCs had a significant reduction in recurrent MI (*P* = 0.04) and trends toward improved mortality, repeat revascularization, and heart failure hospitalizations. When subset analyses were performed, it appeared that improvement in LVEF was more likely among patients with (1) lower baseline LVEF, (2) those in whom cells were infused during the repair phase (days 5–7 after STEMI), (3) in those receiving >10^8^ BMMNCs, and (4) cells prepared without heparin as it appears to reduce function of SDF-1 [26]. 

The composition of mononuclear cells by cell subtype and cell potency may also be important predictors of efficacy. CD34^+^ expressing BMMNCs are enriched for hematopoietic and (to a lesser degree) endothelial progenitor cells and have been shown experimentally to localize more avidly in the peri-infarct zone than unselected mononuclear cells not expressing CD34^+^ epitope [27]. Using radio-labeled cells, it has been demonstrated that a greater proportion of CD34^+^ cells home to peri-infarct zones after intracoronary delivery than undifferentiated BMMNCs [27, 28]. Further, the biologic potency measured as mobility of cells coexpressing CXCR-4 in a stromal derived factor-1 (SDF-1) gradient appears to be a crucial determinant of efficacy [25, 29–31]. Thus, the current shift in post-MI cell therapy field is towards delivering a highly selected and highly mobile progenitor cell product at a sufficient dose to maximize preservation of cardiac function. 

CD34 is a novel hematopoietic progenitor cell antigen that is expressed in human bone marrow, blood, and fetal liver [32, 33]. *In vitro*, CD34^+^ cells differentiate into endothelial- and smooth muscle-like cells and form angiogenesis-like networks in Matrigel [34]. Originally considered the putative “endothelial progenitor cell” (EPC) [35], bone marrow CD34^+^ cells are a relatively uncommon cell type (~1-2%) in human bone marrow and are now known to transdifferentiate into hematopoietic lineage cells and rarely also into endothelial cells, smooth muscle cells, and cardiomyocytes *in vivo* [36]. CD34 antigen is expressed by immature endothelial cell precursors and appears to confer hematopoietic potential, especially in combination with CD133 [33]. In contrast, coexpression of CD34 and the vascular endothelial growth factor receptor-2 denotes a population enriched for endothelial progenitors [37]. 

Stamm et al. first attempted to utilize CD34^+^ cells in humans after MI [38]. In a study of six subjects with a history of MI, dual positive CD34^+^/CD133^+^ cells were injected in the infarct border zone during coronary artery bypass grafting. On the day prior to surgery, subjects underwent a bone marrow harvest from the iliac crest. Marrow aspirate was isolated for mononuclear cell by ficoll density centrifugation followed by monoclonal antibody selection for CD133^+^ cells. These cells showed high purity for CD34^+^ and CD133^+^ markers (75–90%) and high viability (75–91%). Cells were delivered in the operating room during CABG by direct myocardial injection prior to reperfusion. At six-month followup, all patients had improved perfusion by SPECT, and 4 of 6 patients had significantly improved LVEF and diastolic LV dimensions by transthoracic echocardiogram.

The prospect of delivering isolated CD34^+^ cells in favor of unselected cells for post-MI repair garnered attention from a study comparing human CD34^+^ cells to human total mononuclear cells in a nude rat MI model [30]. Animals receiving CD34^+^ cells had greater capillary density in the infarcted myocardium, lower percent fibrosis, and a significantly improved cardiac function at 28 days compared to their counterparts receiving unselected mononuclear cells. 

The REGENT trial addressed the relative efficacy of CD34^+^ cells compared to unselected cells in humans after STEMI [25]. One hundred and sixty patients were randomized to receive either 1.8 × 10^8^ unselected autologous BMMNCs, 1.9 × 10^6^ CD34^+^ CXCR4^+^ cells, or no cells 7 days following STEMI. The two treatment groups had absolute improvement in LVEF by 3% at 6-month followup (*P*s < 0.05), while LVEF in the control group (no cells) did not change (*P* = 0.73). A greater proportion of patients receiving CD34^+^ CXCR4^+^ cells improved LVEF at 6 months (51%), compared to 39% of those receiving unselected cells and 36% for controls; however, this difference did not reach statistical significance. There was no significant difference in major adverse cardiovascular events (MACEs) between the groups at 6 months.

## 4. AMR-001

### 4.1. Hypothesis

We recently completed the first prospective, dose-escalation controlled trial to determine whether a CD34^+^ cell dose threshold for effect exists after acute STEMI [39]. We hypothesized that intracoronary autologous bone marrow-derived CD34^+^ cell infusion would be safe, and the therapeutic effect would be dose dependent. 

### 4.2. Study Design

To determine the effective CD34^+^ cells cell dose threshold, we prospectively assessed the safety and efficacy of intracoronary infusion of autologous bone marrow-derived CD34^+^ cells administered sequentially at three dose levels (5, 10, and 15 millions). Subjects with acute STEMI successfully treated with intracoronary stent implantation within three days of hospitalization were consented. Only patients with LVEF ≤50% by echocardiography and a regional wall motion abnormality in the distribution of the infarct related artery, four or more days after stenting, were enrolled. Subjects were enrolled randomly at each site as controls (*N* = 15) to receive the standard of care or to the open label cell therapy group to receive one of three dose levels of CD34^+^ cells (5, 10, or 15 millions; *N* = 5 in each group).

### 4.3. Methods

From five to eight days after coronary stenting, patients in the treatment group had a minibone marrow harvest using conscious sedation and local anesthesia. Harvested cells were transferred to a central GMP facility (Progenitor Cell Therap, Hackensack, NJ, USA) where CD34^+^ cells were isolated and enriched using the anti-CD34 Mab and Dynabeads (Baxter, Deerfield, IL, USA) on the Isolex 300i system. CD34^+^ cell enumeration, purity, and viability were assayed by flow cytometry using anti-CD34/CD45 antibody and 7-AAD (Stem-Kit reagents, Beckman Coulter, Brea, CA, USA). Endotoxin levels were determined using Limulus Amebocyte Lysate Kinetic-QCL Test Kit (Lonza, Allendale, NJ, USA). Sterility was tested by 14-day product culture in fluid thioglycollate medium and tryptic soy broth. Preliminary product sterility assessment was determined by gram staining. The final CD34^+^ cell product was formulated in 6 mL of Dulbecco's Phosphate Buffered Saline (Baxter) and 4 mL (40%) of autologous human serum containing 1% human serum albumin (Alpha) and 25 USP units/mL of heparin sodium stored in a sterile 10 mL syringe. Selected CD34^+^ cells were aliquoted according to targeted doses and tested for coexpression of VEGFR-2 and CXCR-4 by flow cytometry and assessed *in vitro* for SDF-1 mobility and colony-forming unit-granulocyte-macrophage (CFU-GM) growth as described elsewhere [6, 40]. The percentage of CD34^+^ cells within harvested bone marrow and CD34^+^ cell mobility in an SDF-1 gradient was compared with and without autologous serum in age-matched healthy volunteers free from any symptomatic coronary artery disease (*N* = 10) and patients participating in our trial (*N* = 6). 24–48 hours after bone marrow harvest, treatment group subjects (*N* = 16) underwent repeat coronary angiography, and the cell product was infused via an over-the-wire balloon catheter positioned within the stented segment using a stop-flow technique described previously [6]. 

### 4.4. Results

Cell harvest and infusion were safe. Quantitative rest hypoperfusion score measured by SPECT improved at 6 months in subjects receiving ≥10 million CD34^+^ cells compared with controls (−256 versus +14; *P* = 0.02). There was a trend toward improvement of ejection fraction (+4.5%, *P* = 0.059 compared to baseline) in the high-dose groups compared to no change in controls and those receiving 5 million CD34^+^ cells (+0.7%). Improvement in SPECT perfusion and infarct size reduction correlated with the number of CD34^+^ demonstrating mobility with stromal derived factor-1. 

The apparent dose threshold of infused CD34^+^ was identified in post hoc analysis. In comparison to controls or those receiving low dose of cells, intracoronary infusion of ≥10 million CD34^+^ cells was associated with significant improvement in myocardial perfusion measured by resting single-photon emission computed tomography (SPECT) and further quantified by a resting total severity score (RTSS). RTSS is a composite of the extent and severity of the perfusion defect assessed by SPECT, and serves as a potential index of cardiomyocyte viability that is validated to detect ≥10% difference on repeat measure [41]. Two previous studies that measured infarct region perfusion have observed similar changes [6, 42]. Further supporting a CD34^+^ cell dose threshold was the concomitant trend toward improvement of LVEF among patients receiving ≥10 million CD34^+^ cells, whereas patients receiving either 5 million CD34^+^ cells or the controls had no change. This was despite the fact that patients who received ≥10 million CD34^+^ cells had larger infarct sizes and greater RTSS at baseline that would have predisposed them to a greater likelihood for adverse ventricular remodeling [43]. Moreover, the continued improvement in LVEF between 3 months and 6 months in patients who received ≥10 million CD34^+^ cells, that was not seen in controls, suggests a progressive therapeutic effect that is consistent with recovery from hibernation and reduced apoptosis, a process that may be delayed for up to 6 months [44, 45].

### 4.5. Conclusion

We concluded that intracoronary infusion of selected bone marrow-derived CD34^+^ cells during the repair phase after STEMI is safe, and at a dose threshold of ≥10 million CD34^+^ cells, it is associated with a significant improvement in perfusion that may limit deterioration in cardiac function. 

## 5. PreSERVE-AMI

To followup our Phase I clinical trial with the bone marrow-derived autologous CD34^+^ cell product, we have launched the PreSERVE-AMI trial, a Phase II multicenter, randomized, double-blind, placebo-controlled clinical trial to evaluate the efficacy and safety of AMR-001 at a dose of ≥10 × 10^6^ CD34^+^ cells. The trial will enroll 160 patients with STEMI and reduced LVEF (<48% by cardiac magnetic resonance imaging 96 hours after stent placement). Subjects will be randomized 1 : 1 to treatment and placebo arms.

The primary aim of the PRESERVE-AMI is to demonstrate safety and determine the effect of intracoronary delivery of AMR-001 on myocardial perfusion by the resting total severity score (RTSS), as measured by gated SPECT MPI at baseline and 6 months. The secondary objectives are to assess the effect of AMR-001 on infarct size and cardiac function (LVEF), end systolic and end diastolic volumes, regional myocardial strain, and regional wall motion by CMR measured at baseline and at 6 month followup. Additionally, the impact of AMR-001 on quality of life (QOL) indices and clinical outcomes will be determined. QOL will be measured by the Kansas City Cardiomyopathy Questionnaire (KCCQ) and the Seattle Angina Questionnaire (SAQ) which will be administered at baseline, 6, and 12 months after randomization. Clinical outcomes, including MACE and change in NYHA class at 6 months, 12 months, 18 months, 2 years, and 3 years, will be monitored. MACE will be defined as cardiac death, hospitalization for worsening heart failure, and recurrent myocardial infarction. Clinical events such as ventricular arrhythmias requiring intervention, acute coronary syndrome (ACS), and coronary revascularization will be assessed as the same time points (6 months, 12 months, 18 months, 2 years, and 3 years). Additionally, total days alive and total days outside the hospital will be determined at 6 and 12 months. Tertiary objectives include characterizing the relationship between the quantity and quality of infused cells and perfusion, infarct size, LVEF, and clinical outcomes. We further aim to correlate initial predictors of outcomes including baseline LVEF, baseline infarct size, baseline RTSS score, number of prior AMI, IRA site, and time from STEMI to stent placement with changes in perfusion, infarct size, LV function, and clinical outcomes. Anticipated date of completion for the primary aim is January, 2013.

## 6. Conclusion

Intracoronary delivery of bone marrow-derived mononuclear cells by the “stop-flow technique” after acute STEMI has proven to be safe and is associated with modest improvement in LVEF [6–8, 10, 13, 15–17, 20–23, 25, 29, 30, 39, 46–48]. Meta-analyses suggest that the benefit of cell therapy on postinfarct cardiac function is only apparent if cells are infused during the repair phase after STEMI, given in sufficient quantity, and with adequate mobility in an SDF-1 gradient [29, 31, 47]; however, a major limitation has been the heterogeneity of unselected bone marrow mononuclear cells and the variability in cell doses employed. The CD34 surface marker identifies a population of cells within the bone marrow that exhibit regenerative characteristics, but only one study (in addition to our own) has examined the utility of CD34^+^ cell therapy, but at a relatively low cell dose [25, 49]. Our recently published Phase I trial is the first prospective, dose-escalation controlled trial to determine if a dose threshold for effect exists. We demonstrated the apparent safety of harvesting 320 mL of autologous bone marrow from 5 to 8 days after STEMI, and successfully generated a sterile CD34^+^ stem cell product. 

Whereas most unselected mononuclear cell therapy studies have infused doses of CD34^+^ cells that were consistent with our lowest dose cohort [6, 10, 13, 15–17, 19–23, 46], only one utilized doses comparable to the ≥10 million cell cohorts in our study [13] (Table 1). Sustained improvement in LVEF was only observed in those receiving the higher cell doses, a finding also reported in recent meta-analyses [7, 8, 15, 29, 50].

Natural mobilization of CD34^+^ cells coexpressing CXCR4 that home to ischemic regions in response to an SDF-1 gradient induced by nuclear localization of hypoxia inducible factors after MI predicts prevention of cardiomyocyte loss and preservation of LVEF [31, 51]. Prevention of cardiomyocyte apoptosis and rescue from hibernation via paracrine effects (including AKt activation), as well as increased microvascular perfusion via neoangiogenesis, appear to underlie the restorative effects of infused CD34^+^ cells that appear to preserve cardiac function for up to four years in addition to lower adverse long-term event rate [18, 47, 48, 52].

One of the vital findings from our Phase I trial is that product potency (in terms of improvement in perfusion and LVEF) was related to the mobility of CD34^+^ cells in an SDF-1 gradient, as previously reported [6, 29]. Furthermore, cell mobility declined over time following bone marrow harvest, with a median 57% decrease between 24 and 48 hours after harvest and a further 11% decline by 72 hours. Administration of cells without delay after harvest may reduce the quantity of cells required for the therapeutic effect [53, 54]. Thus, the number of CD34^+^ cells, their SDF-1 mobility, and the time from harvest to infusion are all factors that appear to determine potency of the cell product.

The discrepancy between positive animal studies and mixed clinical trials, as is often the case, requires further investigation by larger, well-controlled trials with adequate followup. The previous decade has taught us that bone marrow harvesting and intracoronary infusion of bone marrow-derived CD34^+^ cells in doses up to 15 million cells during the repair phase after STEMI are feasible and safe. Yet, the interpretation of the benefit of CD34^+^ cell product as an ancillary treatment for acute STEMI is limited by the small number of studies, incomplete followup results, and significant variability in measures of cardiac function. The PreSERVE-AMI trial, that is currently underway, serves as an important “next step” toward addressing these issues and identifying a clinically viable cell product for the post-PCI management of STEMI. 

## Figures and Tables

**Table 1:** Clinical trials of bone marrow cell (BMC) therapy by intracoronary delivery following acute ST-segment elevation MI (STEMI).

Study author (trial name)	Date published	*N*	Days after STEMI (mean)	Primary outcome	Mean no. CD34^+^cells (millions)
Assmus et al., [10] (TOPCARE-AMI)	2002	20	4	Improved LVEF	7.4
Fernandez-Aviles et al., [46]	2004	20	13	No difference	Not reported
Bartunek et al., [55]	2005	35	11.6	Improved LVEF	15.4
Ge et al., [15] (TCT-STAMI)	2006	20	<1	Improved LVEF	0.1
Hirsch et al., [19] (HEBE)	2011	200	5	No difference	4
Huikuri et al., [16] (FINCELL)	2008	80	3	Improved LVEF	2.6
Janssens et al., [20] (LEUVEN-AMI)	2006	67	<1	No difference	2.8
Lunde et al., [21] (ASTAMI)	2006	97	6	No difference	0.7
Meluzín et al., [50]	2008	60	6.9	Improved LVEF	Not reported
Quyyumi et al., [39] (AMR-001)	2011	31	8	Positive trend towards improved EF in highest dose group	5, 10, 15
Roncalli et al., [56]	2011	101	9.3	Improved myocardial viability	1.2
Schachinger et al., [17] (REPAIR-AMI)	2006	204	4	Improved LVEF	3.6
Strauer et al., [6]	2002	10	7	No difference	<0.6
Tendera et al., [25] (REGENT)	2009	200	7	No difference	1.9 (CD34^+^ CXCR4^+^ cell group), not reported for unselected cell group
Traverse et al., [23]	2010	40	5	No difference	1.6
Traverse et al., [22] (LateTIME)	2011	87	17	No difference	3.8
Wollert et al., [13] (BOOST)	2004	60	5	Improved LVEF	9.5
